# Migraine-relevant sex-dependent activation of mouse meningeal afferents by TRPM3 agonists

**DOI:** 10.1186/s10194-021-01383-8

**Published:** 2022-01-10

**Authors:** G. Krivoshein, E. A. Tolner, AMJM van den Maagdenberg, R. A. Giniatullin

**Affiliations:** 1 A.I.Virtanen Institute for Molecular Sciences, University of Eastern Finland, Kuopio, Finland; 2 Department of Human Genetics, Leiden University Medical Center, Leiden, The Netherlands; 3 Department of Neurology, Leiden University Medical Center, Leiden, The Netherlands; 4 Laboratory of Neurobiology, Kazan Federal University, Kazan, Russia

**Keywords:** Migraine, TRPM3, Nociception, Sex-dependence, Pregnenolone sulfate, CIM0216

## Abstract

**Background:**

Migraine is a common brain disorder that predominantly affects women. Migraine pain seems mediated by the activation of mechanosensitive channels in meningeal afferents. Given the role of transient receptor potential melastatin 3 (TRPM3) channels in mechanical activation, as well as hormonal regulation, these channels may play a role in the sex difference in migraine. Therefore, we investigated whether nociceptive firing induced by TRPM3 channel agonists in meningeal afferents was different between male and female mice. In addition, we assessed the relative contribution of mechanosensitive TRPM3 channels and that of mechanosensitive Piezo1 channels and transient receptor potential vanilloid 1 (TRPV1) channels to nociceptive firing relevant to migraine in both sexes.

**Methods:**

Ten- to 13-week-old male and female wildtype (WT) C57BL/6 J mice were used. Nociceptive spikes were recorded directly from nerve terminals in the meninges in the hemiskull preparations.

**Results:**

Selective agonists of TRPM3 channels profoundly activated peripheral trigeminal nerve fibres in mouse meninges. A sex difference was observed for nociceptive firing induced by either PregS or CIM0216, both agonists of TRPM3 channels, with the induced firing being particularly prominent for female mice. Application of Yoda1, an agonist of Piezo1 channels, or capsaicin activating TRPV1 channels, although also leading to increased nociceptive firing of meningeal fibres, did not reveal a sex difference. Cluster analyses of spike activities indicated a massive and long-lasting activation of TRPM3 channels with preferential induction of large-amplitude spikes in female mice. Additional spectral analysis revealed ​a dominant contribution of spiking activity in the α- and β-ranges following TRPM3 agonists in female mice.

**Conclusions:**

Together, we revealed a specific mechanosensitive profile of nociceptive firing in females and suggest TRPM3 channels as a potential novel candidate for the generation of migraine pain, with particular relevance to females.

## Introduction

Migraine is a common multifactorial brain disorder with a prevalence of approximately 15% [1, 2]. Migraine is typically characterized by recurrent attacks of severe, often unilateral pulsating headache accompanied by nausea, vomiting and/or photo- and phonophobia [3]. Three times more women than men are affected. The sex difference is thought to be due to fluctuations in female sex hormones, as evidenced by observations that the prevalence of migraine strongly increases in women after menarche and attack frequency changes during pregnancy and menopause [4]. One proposed mechanism is that a drop in the level of estrogen around menstruation would lead to increased brain excitability, thereby triggering the trigeminovascular system [5, 6]. Activation of the trigeminovascular system involves firing of trigeminal (TG) neurons that innervate the meninges [7–10]. We showed earlier that TG neurons express mechanosensitive Piezo1 channels that are activated by specific agonist Yoda1 [11, 12]. In addition to Piezo1 channels, other nociceptors are likely involved in meningeal TG firing, such as non-mechanosensitive TRPV1 channels [13]. Here we propose that mechanosensitive ‘transient receptor potential melastatin 3’ (TRPM3) channels, which were recently shown to be expressed in human sensory neurons [14], are involved in the generation of migraine pain. Their functional role in nociception is apparent given that TRPM3 channels sense noxious heat and TRPM3 deficient mice show reduced inflammatory pain [15]. Of relevance to migraine pathophysiology, TRPM3 channels co-localize in TG neurons with the nociceptive fibre neuropeptide calcitonin gene-related peptide (CGRP) [16], of which plasma levels were shown to be elevated during migraine headache [17] and antagonism of CGRP has proven effective in treating migraine [18]. Most relevant to our study, TRPM3 channels respond to endogenous neurosteroid pregnenolone sulfate (PregS) [19], and TRPM3 channel activation can be suppressed by sex hormones progesterone and 17β-oestradiol [20]. Therefore, one can speculate that regulation of TRPM3 channels by sex hormones might, in fact, represent an endogenous inhibitory mechanism that modulates migraine attacks in females. Of note, the non-steroidal anti-inflammatory drug diclofenac and anticonvulsant primidone are highly efficient blockers of TRPM3 channels [21]. Therefore, TRPM3 channels have been proposed as a clinically promising pharmacological target for analgesic strategies [22], although this has not been considered yet for migraine. Here we show that TRPM3 channels are present in the meningeal part of the trigeminovascular system and can play a particular role in the generation of migraine pain in females.

## Material and methods

### Animals

Experiments were performed in 10- to 13-week-old male and female wildtype (WT) C57BL/6 J mice. Mice were bred in the Animal Facility of the University of Eastern Finland (UEF) and housed in special cages in rooms with controlled temperature 22 °C, humidity, and a 12-h light/dark cycle. Food and water were provided ad libitum. All experimental procedures were performed following the ethical guidelines of the European Community Council Directive of 22 September 2010 (2010/63/EEC). The study protocol was approved by the Animal Care and Committee of the University of Eastern Finland (licence EKS-008-2019, protocol from 25 November 2019). All measures were taken to minimize animal suffering in accordance with ARRIVE guidelines.

### Hemiskull preparation and solutions

Isolated mouse hemiskull preparations for direct spike recordings from TG nerve endings were prepared as previously described [13, 23]. In brief, after CO_2_ inhalation and checking for lack of a pedal withdrawal reflex, mice were sacrificed by cervical dislocation followed by decapitation. Subsequent cleaning procedures were carried out for 15–20 min in oxygenated artificial cerebrospinal fluid (aCSF), containing (in mM): 120 NaCl, 2.5 KCl, 2 CaCl_2_, 1 MgCl_2_, 11 glucose, 24 NaHPO_4_ and 30 NaHCO_3_, bubbled with 95% O_2_/5% CO_2_ at room temperature (RT), while pH was maintained at 7.25–7.35. Skin and cranial muscles were removed from the outer side of the skull, which was then divided into two parts along the sagittal line using a scissors. To provide access for the recording electrode to the meningeal nerves branching out from the TG ganglia, the brain was gently removed with a forceps without harming the TG ganglia. Special attention was paid to keep the dura mater with meningeal nerves and vessels on the bone tissue inside the hemiskull (virtually) intact. After that, the isolated hemiskull was placed in a recording chamber continuously perfused with aCSF (6–7 mL/min) and oxygenated with 95% O_2_/5% CO_2_ mixture.

Drugs were purchased from Tocris Bioscience, UK, i.e. TRPM3 channel agonists pregnenolone sulfate (PregS) and CIM0216, Piezo1 agonist Yoda1 and transient receptor potential vanilloid 1 (TRPV1) channel agonist capsaicin and dissolved in DMSO. Substances were diluted to a final concentration in aCSF immediately before usage and applied to the receptive field around the main meningeal branch of the TG nerve by fast perfusion (~ 6 mL/min).

### Electrophysiological recordings

To detect TRPM3 mechanosensitive channels in TG nerves of meningeal tissue, we employed direct electrophysiological spike recording of nociceptive firing from peripheral meningeal nerve terminals. In the preparation stage, after placing the hemiskull preparation into the recording chamber, the main meningeal branch of the TG nerve was cleaned from surrounding tissue and cut at a distance of ~ 0.5 mm from the TG ganglion. Next, a small incision was made in the dura mater, and the peripheral part of the cut meningeal branch was placed into a recording glass microelectrode filled with aCSF. The exposed tip of the microelectrode was adjusted to the nerve diameter to allow the nerve ending to entirely plug it. Next, a silver reference electrode was placed into the bath containing the hemiskull preparation. At the start of each experiment, to allow the nerve to be tightly sucked into the electrode and stabilize the baseline, 10 min of spontaneous spike activity was recorded as control. After baseline recording, 50 mM KCl with compensated osmolarity was applied to verify the neuronal activity of the preparation. Next, to demonstrate the presence of mechanosensitive fibres, mechanosensitive Piezo1 channels [12] were activated with Yoda 1 (5 μM) whereas TRPM3 mechanosensitive channels were activated with PregS (50 μM) or CIM0216 (5 μM). Finally, as a marker of non-mechanosensitive nociceptive neuronal activity mediated by TRPV1 receptors, capsaicin (1 μM) was applied. Note that all drug applications were made to the same peripheral terminal of the meningeal nerve and lasted for 10 min with subsequent perfusion for 20 min (washout). Because Yoda1, PregS, CIM0216 and capsaicin were all prepared in DMSO, a pre-application of vehicle solution containing the same concentration of DMSO was administered, which did not significantly affect spiking activity of the TG nerve.

Electrophysiological recordings of neuronal spiking activity generated in the peripheral part of the meningeal nerves were registered at RT using a low-noise digital amplifier (ISO-80; World Precision Instruments, Sarasota, FL, USA) with the following parameters: gain 10,000X and bandpass 300–3000 Hz. Obtained electrical signals were digitized at 8-μsec intervals using a NIPCI 6221 data acquisition board (National Instruments, Austin, TX, USA) and stored on a PC for offline analysis. Signals were visualized by WinEDR v.3.5.2 software (University of Strathclyde, Glasgow, UK) and analyzed with MATLAB-based software (MathWorks, Natick, MA, USA) [13].

### Cluster and spectral analysis of spiking activity

Advanced cluster and spectral spike analysis was performed, as described previously [13, 24, 25]. In brief, before analysis, all original recordings were filtered at 100–9000 Hz using a Chebyshev type 2 filter for spike detection and subsequent cluster and spectral analysis. The baseline noise level was estimated at the beginning of each experiment in a spikeless interval lasting at least 20 s to determine the respective threshold for spike detection. A recording was considered to contain spikes when the potential amplitude exceeded 5 standard deviations (SD) of the level of baseline noise. Spike amplitudes were normalized by baseline noise and expressed as SD values (arbitrary units, a.u.). Two-phase signals with a duration in the range of 0.3–1.8 ms were considered to represent action potentials, for which the spike rate was presented as number of spikes per 10 s. Using MATLAB software, for each spike, we calculated parameters such as the rise and decay time, the amplitude of positive and negative phases, spike areas, and their total duration. The ‘KlustaKwik’ application [26] was used to automatically recognize the most compact groups of spikes (clusters). Amplitudes of positive and negative phases of spikes were used as input parameters for clusterization. This approach allowed us to separate the total flow of spikes into 7 to 30 individual clusters for each experiment. For spectral analysis, data were analyzed with respect to the number of interspike intervals (ISI) per second, both for the whole nerve and for spike activity of individual clusters.

### Data analysis and statistics

Electrophysiological data were analyzed and plotted using Origin Pro (Origin Lab Corporation, Northampton, MA, USA) and Graph Pad Prizm (GraphPad Prizm Software, La Jolla, CA, USA). We used at least five independent replicates for each set of experiments, where n corresponds to the number of animals. The resulting data were presented as the mean ± standard error of the mean (m ± SEM). The significance level was set at *p* < 0.05, statistically assessed by the Wilcoxon signed-rank test for paired data and the Mann–Whitney U test for unpaired data.

## Results

### Distinct mechanosensitive activation of trigeminal nerve terminals in male and female mouse meninges

**Fig. 1:**
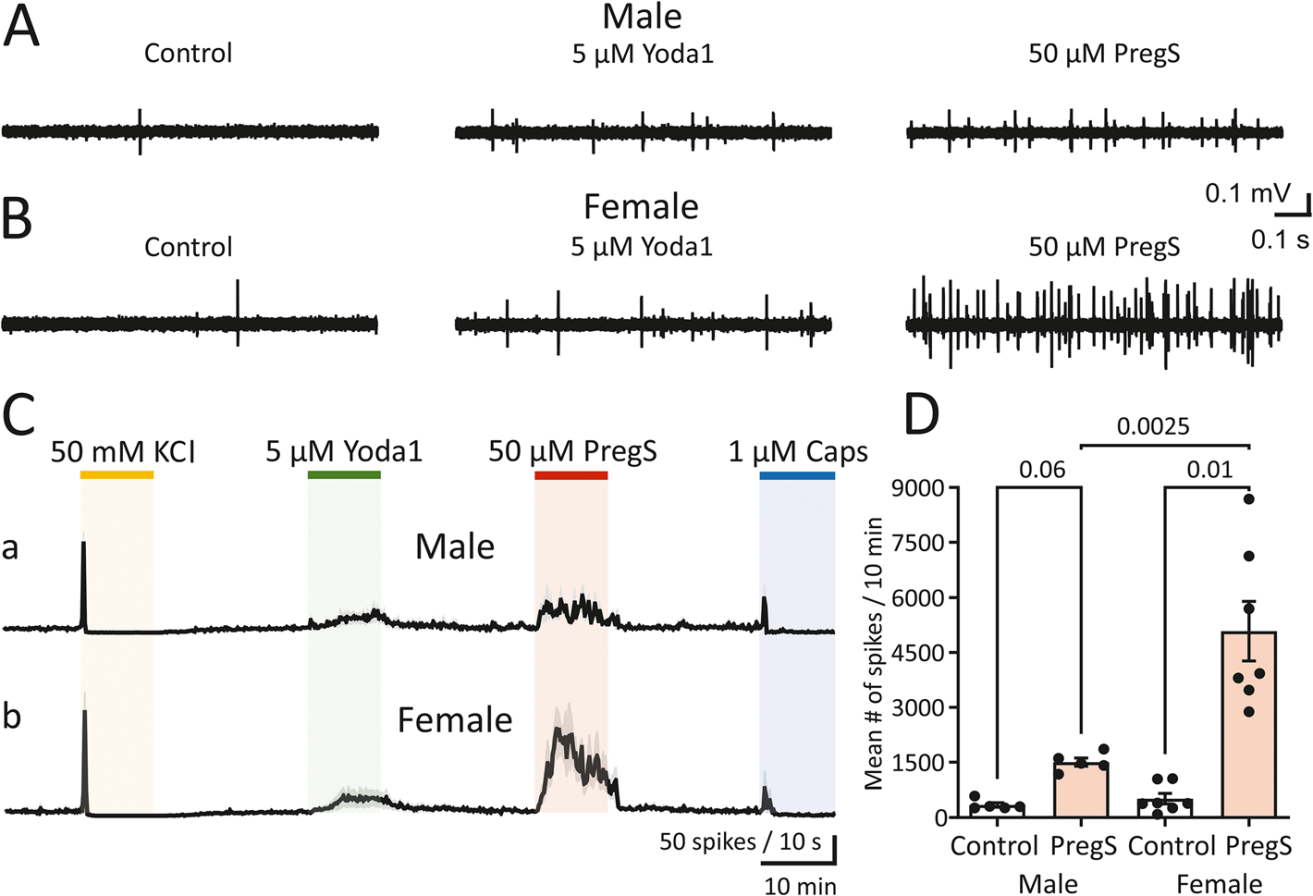
Activation of trigeminal nerve terminals by PregS in male and female mouse meninges. (**A, B**) Example traces of multi-unit activity (MUA) in the TG nerve innervating meninges in a hemiskull preparation of male (**A**) and female(**B**) mice recorded in the control condition (left), after application 5 μM Yoda1 (middle) and after 50 μM PregS (right). (**C**) Time course of spike frequency (10-s bin size) induced by application of 50 mM KCl, 5 μM Yoda1, 50 μM PregS and 1 μM capsaicin in males and females (mean ± SEM, *n* = 5 and *n* = 7, respectively). Notice the stable recovery of persistence firing during a 20-min washout after each drug application. (**D**) Histograms show the mean number of nociceptive spikes during 10-min recordings before (control) and after application of 50 μM PregS in males (mean ± SEM, *n* = 5, *p* = 0.06, Wilcoxon signed-rank test) and females (mean ± SEM, *n* = 7, *p* = 0.01, Wilcoxon signed-rank test). Notice the sex difference in firing when 50 μM PregS was applied (*p* = 0.0025, Mann–Whitney U test)

To assess possible involvement of two types of mechanosensitive channels in the activity of meningeal TG nerve fibres in male and female mice, first 50 mM KCl was applied for 10 min to measure overall excitability of the preparation. Then, after washout of KCl, sequentially 5 μM Yoda1 (as Piezo1 agonist), 50 μM PregS (as TRPM3 agonist) and 1 μM capsaicin (as agonist of TRPV1 receptors, typically expressed in nociceptors) were tested in the same preparation. Examples of the firing activity of the nerve fibres before and after application of Yoda1 and PregS in male and female meningeal hemiskull preparations are shown in Fig. 1 A, B. Application of Yoda1 led to a moderate induction of persistent firing - compared to the spike activity during the 10 min control recording period directly prior to drug application (Fig. 1A and C) - with no difference between males (from 346.9 ± 73.9 prior to 1367 ± 297.3 during Yoda1) and females (from 370.2 ± 97.5 prior to 1507 ± 338.4 during Yoda1; *p* = 0.76, Mann–Whitney U test). In contrast, PregS application led to profoundly increased firing in females, namely from 515.1 ± 143.1 to 5084 ± 815.4 (*n* = 7, *p* = 0.01, Wilcoxon signed-rank test), while in males the observed increase in firing during PregS (from 343.2 ± 61.31 prior to 1503 ± 112.9) was not significant (*n* = 5, *p* = 0.06, Wilcoxon signed-rank test; Fig. 1 B-D). Thus, in females, PregS induced a much stronger increase in spiking activity than in males (*p* = 0.0025, Mann–Whitney U test; Fig. 1 C, D). The amplitude of action potentials in basal conditions did not show sex difference (7.6 ± 0.5 a.u. in females; *n* = 7 vs. 6.6 ± 0.4 a.u. in males; *n* = 5. *p* = 0.14; Mann–Whitney U test). However, there was a sex difference in amplitude of action potentials during PregS application which were larger in females (13.8 ± 1.2 a.u. in females; *n* = 7 vs. 8.9 ± 0.4 a.u. in males; *n* = 5, *p* = 0.01; Mann–Whitney U test).

### Lack of interaction of Piezo1 and TRPM3 channels during sequential activation

**Fig. 2:**
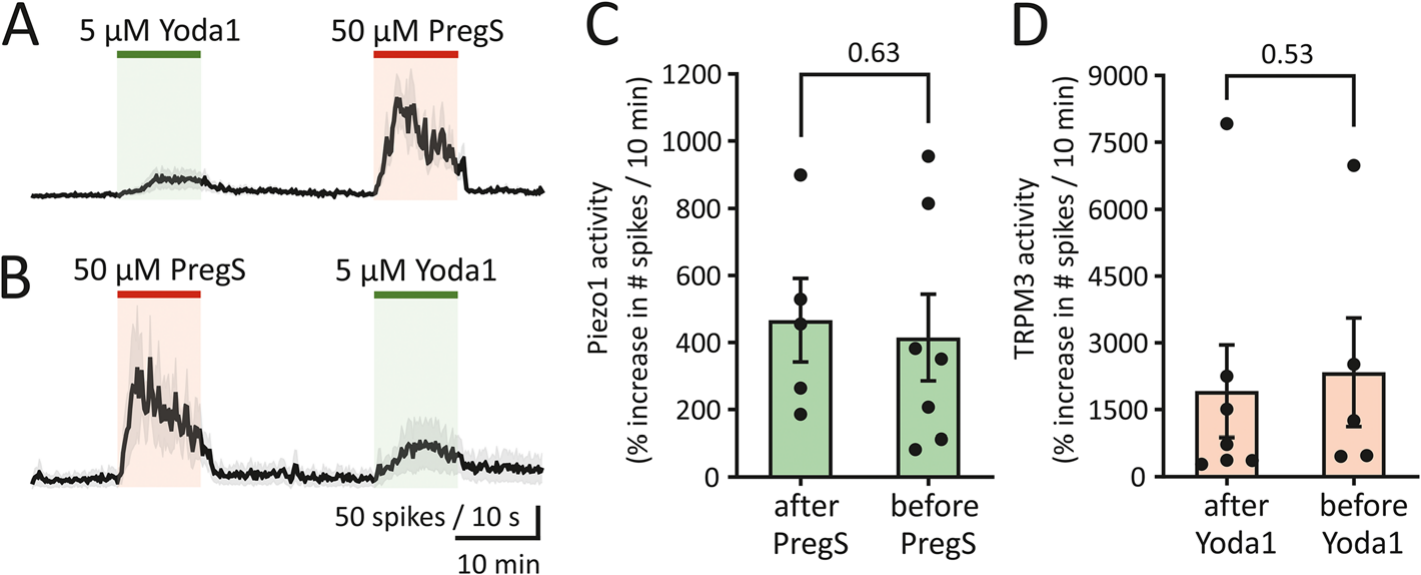
Activation of Piezo1 channels has no impact on increased nociceptive firing during PregS application. (**A**) Time course of MUA in meningeal nerves of female mice when first 5 μM Yoda1 and then 50 μM PregS was applied (mean ± SEM, *n* = 7). **(B)** Time course of MUA in female mice when first 50 μM PregS and then 5 μM Yoda1 was applied (mean ± SEM, *n* = 5). **(C)** Histograms show the percentage of increased nociceptive firing in females during 10-min recordings following 50 μM PregS application when administered before (mean ± SEM, n = 5) and after 5 μM Yoda1 application (mean ± SEM, *n* = 7). Notice that there is no difference in the proportion of increased nociceptive firing between the different orders of drug application (*p* = 0.63, Mann–Whitney U test). **(D)** Histograms show the percentage of increased nociceptive firing in females during 10-min recordings following 5 μM Yoda1 application when administered before (mean ± SEM, *n* = 7) and after 50 μM PregS application (mean ± SEM, *n* = 5). Notice there is no difference in the fraction of increased nociceptive firing between the different orders of drug application (*p* = 0.53, Mann–Whitney U test)

The presence of two types of mechanosensitive channels in meningeal afferents, as detected during their sequential activation by Piezo1 and TRPM3 agonists, raises the issue whether or not the two channels interact, and if so, whether they promote or depress effects of the other. A functional calcium-dependent interaction has been presented for Piezo and TRPV1 channels [11]. As Piezo1 and TRPM3 mechanosensitive channels are both calcium-permeable [11, 27], one can envisage that they may also be able to provide sensitisation when activated after each other. To test for a possible interaction between Piezo1 and TRPM3 channels, the sequence of agonist application was swapped in female mouse meningeal preparations (Fig. 2 A, B). Spiking activity was calculated as the ratio between the number of spikes during the 10 min with the compound present, and the number of spikes during the 10-min period directly before compound application *(*i.e. control), the latter taken as 100%. When PregS was applied after Yoda1, firing induced by activation of TRPM3 channels by PregS increased to 1917 ± 1037% from the control level (*n* = 7; Fig. 2 C). Applying PregS before Yoda1 increased firing induced by activation of TRPM3 channels by PregS to 2339 ± 1221% from the control level (*n* = 5; Fig. 2 C), which was similar (*p* = 0.63, Mann–Whitney U test) to the alternate order of application. When Yoda1 was applied before PregS, the firing induced by activation of Piezo1 channels by Yoda1 increased to 415 ± 129.5% (*n* = 7; Fig. 2 D), while it increased to 466.8 ± 124.8% (*n* = 5; Fig. 2 D) when Yoda1 was applied after PregS. Thus, also for Yoda1 the spike frequencies were similar (*p* = 0.53, Mann–Whitney U test) regardless of the order of application of the agonists. This indicates a lack of interaction (including sensitization) between TRPM3 and Piezo1 channels when sequentially activated in female mouse meninges.

### Profound activation of trigeminal nerve terminals by CIM0216 in females

**Fig. 3:**
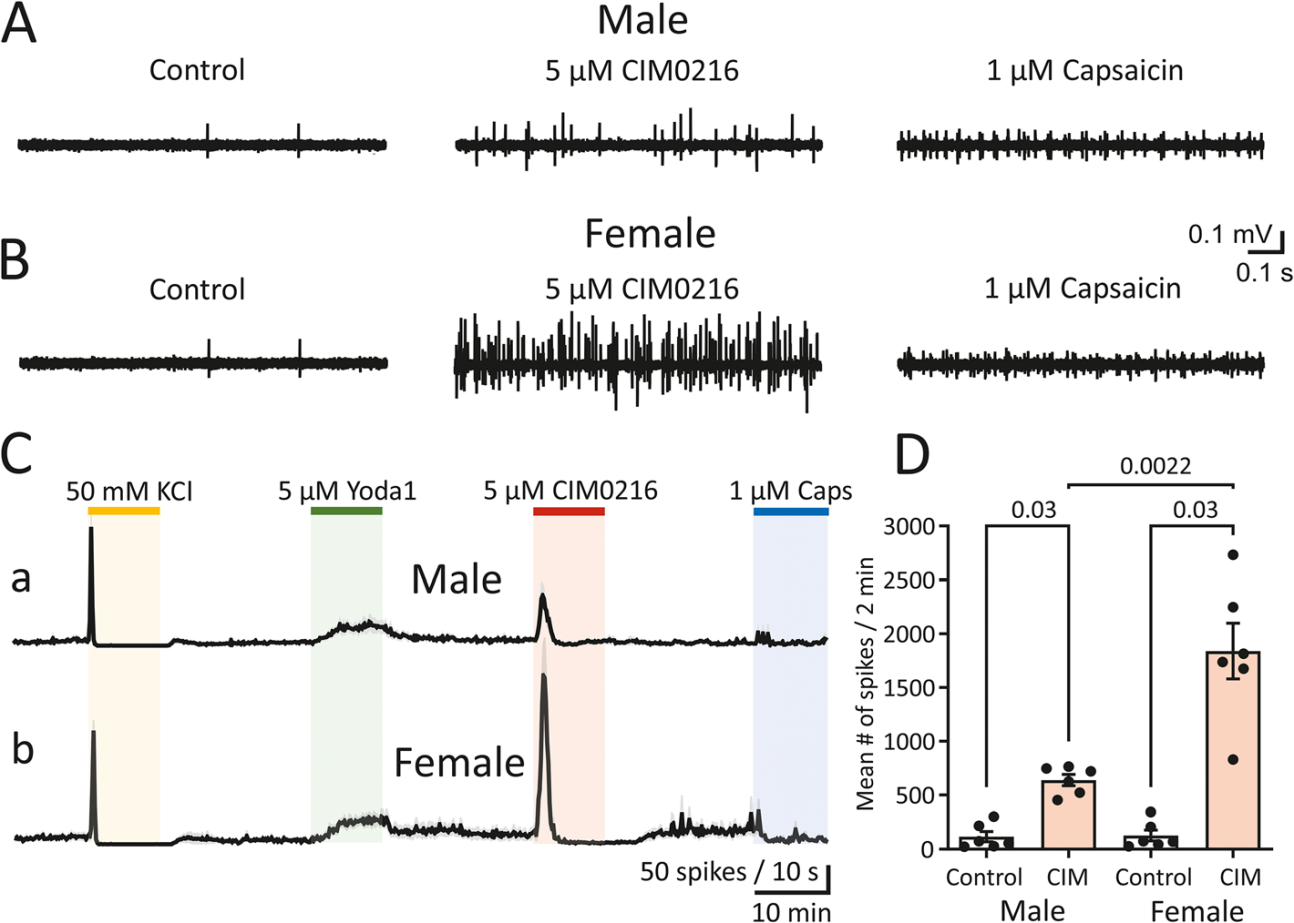
Activation of trigeminal nerve terminals by CIM0216 in male and female mouse meninges. **(A, B)** Example traces of MUA in TG nerves of male **(A)** and female **(B)** mice recorded in the control condition (left), after application 5 μM CIM0216 (middle) and after 1 μM capsaicin (right). **(C)** Time course of spike frequency induced by application of 50 mM KCl, 5 μM Yoda1, 5 μM CIM0216 and 1 μM capsaicin in males and females (mean ± SEM, 10-s bin size, *n* = 6 for both sexes). Notice the difference in the 2-min active phase of 5 μM CIM0216 that was much more pronounced in females, and the subsequent silent time in males compared to overactivated firing recovery during the 20-min washout period in females. **(D)** Histograms show the mean number of nociceptive spikes during 10-min recordings in the control condition and after application of 5 μM CIM0216 in males (mean ± SEM, *n* = 6, *p* = 0.03, Wilcoxon signed-rank test) and females (mean ± SEM, *n* = 6, *p* = 0.03, Wilcoxon signed-rank test). Notice the sex difference in firing when 5 μM CIM0216 was applied (*p* = 0.0022, Mann–Whitney U test)

To confirm our finding that TRPM3 channels are expressed in meningeal afferents, we tested the potent, selective synthetic TRPM3 agonist, CIM0216 [27]. Following the KCl and Yoda1 application we now applied 5 μM CIM0216 instead of PregS. Figure 3 A, B show example traces of spiking activity in meningeal TG nerve fibres from male and female mice in the control condition, during application of 5 μM CIM0216, and after 1 μM capsaicin. Figure 3 C, D shows that 5 μM CIM0216 raised the number of spikes during a 2-min window from 115.8 ± 47.2 during the control condition to 638.3 ± 52.5 in males (*n* = 6, *p* = 0.03, Wilcoxon signed-rank test) and from 127.0 ± 50.6 to 1839.0 ± 259.4 in females (*n* = 6, *p* = 0.03, Wilcoxon signed-rank test). Hence, like PregS, CIM0216 induced profound firing activity in TG nerve terminals, especially in female mice (*p* = 0.0022, Mann–Whitney U test; Fig. 3 C, D). Note that following CIM0216 application, the response to capsaicin was strongly impaired (Fig. 3 A-C) in both males and females, as compared to that observed following PregS application (Fig. 1 C).

### Selective sex-dependence of TRPM3-mediated nociceptive firing with no sex difference in general excitability, Piezo1-mediated activity or capsaicin sensitivity

**Fig. 4:**
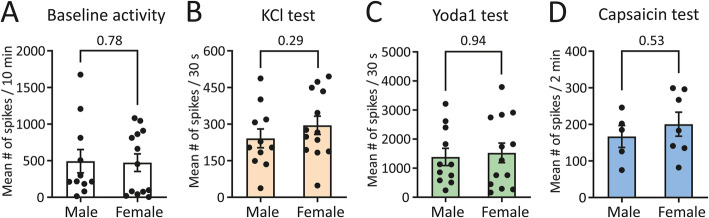
No sex-difference in general nociceptive fibre excitability, Piezo1 activity and capsaicin sensitivity. **(A)** Histograms show the mean ± SEM number of nociceptive spikes in control conditions in males (*n* = 11) and females (*n* = 13) and the lack of a sex difference (*p* = 0.78, Mann–Whitney U test). **(B)** Histograms show the mean ± SEM number of nociceptive spikes during the 30-s active phase of 50 mM KCL application in males (*n* = 11) and females (*n* = 13) and the lack of a sex difference (*p* = 0.29, Mann–Whitney U test). **(C)** Histograms show the mean ± SEM number of nociceptive spikes during 10-min recordings after application of 5 μM Yoda1 in males (*n* = 11) and females (*n* = 13). **(D)** Histograms show the mean ± SEM number of nociceptive spikes during the 2-min active phase of 1 μM capsaicin application in males (*n* = 5) and females (*n* = 7). Notice the lack of a sex difference on firing when 5 μM Yoda (*p* = 0.94, Mann–Whitney U test) and 1 μM capsaicin (*p* = 0.53, Mann–Whitney U test) was applied

One key question is whether the observed sex difference in TG nociceptive firing is exclusively seen with TRPM3 channel activation or whether it is also observed for other nociceptive receptors and/or may relate to non-specific differences in neuronal excitability. To this end, we directly compared the general excitability features (as assessed with KCl application), effects of Piezo1 receptors (as assessed by Yoda1 application), and the capsaicin-induced firing of TG nerve terminals from the experiments from males vs. females. Figure 4 A, B shows the contribution of sex to the overall level of nociceptive neuronal firing by comparing the level of baseline firing to that after application of 50 mM KCl, for both sexes. Notably, the mean number of spikes during a 10-min window at baseline was similar (*p* = 0.78, Mann–Whitney U test) between males and females, i.e. 492.5 ± 158.9 (*n* = 11) and 472.5 ± 120.2 (*n* = 13), respectively. Following KCl application, the mean number of spikes within short burst of activity during the 30-s active phase was also similar (*p* = 0.29, Mann–Whitney U test) for both sexes, i.e. 241.4 ± 38.6 (*n* = 11) in males and 295.0 ± 37.5 (*n* = 13) in females. For the effect of Yoda1, the combined data (expressed as the mean number of spikes per 10 min) from all experiments during Yoda1 application revealed no sex difference for mechanosensitive Piezo1 channels (Fig. 4 C), with the mean number of spikes in males (1367.0 ± 297.3 (*n* = 11)) and females (1507.0 ± 338.4 (*n* = 13)) being similar (*p* = 0.94, Mann–Whitney U test). In addition, the mean number of spikes within the 2-min active phase of application of 1 μM capsaicin in males (166.8 ± 30 (*n* = 5)) and females (200.4 ± 32.9 (*n* = 7)) was also similar (*p* = 0.53, Mann–Whitney U test; Fig. 4 D). Notice that in the last comparison, data with 1 μM capsaicin were taken only from experiments with prior PregS application, as application of 5 μM CIM0216 had an impact on subsequent compound applications. Therefore, a lack of a sex difference for Piezo1 and TRPV1 channels indicates a specific role of sex in the regulation of mechanosensitive TRPM3 channels.

### Cluster analysis of nociceptive spiking activity induced by Yoda1, PregS and capsaicin

**Fig. 5:**
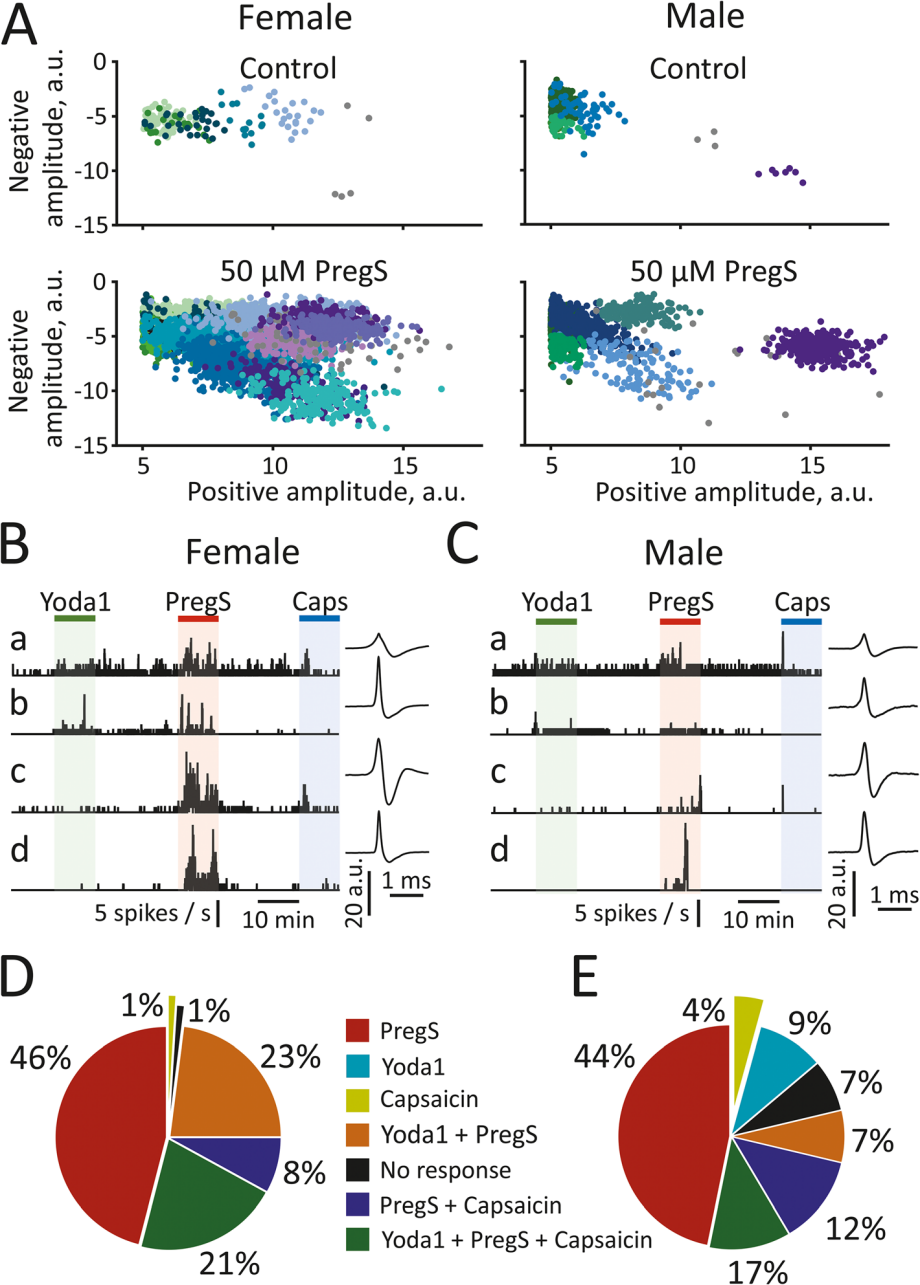
Co-activation of TRPM3 mechanosensitive receptors, Piezo1 mechanosensitive receptors, and TRPV1 channels. (**A**) Presentation of spike clusters within one experiment. Spikes are plotted by separating negative vs. positive amplitude in the control condition (top panels) and after 50 μM PregS application (bottom panels) in females (left) and males (right). Each individual dot indicates a single spike. Dots with similar colours represent one cluster (i.e. representing one fibre or one group of spikes, as separated by the KlustaKwik method. **(B, C)** Examples of clusters within one nerve of female (**B**) and male (**C**) meninges that were sequentially activated by all three agonists (**Ba, Ca**), both 5 μM Yoda1 and 50 μM PregS (**Bb, Cb**) or both 50 μM PregS and 1 μM capsaicin (**Bc, Cc**), or only 50 μM PregS (**Bd, Cd**). Spike shapes are depicted for each cluster. **(D, E)** Pie diagrams demonstrate the averaged percentage of clusters with various neurochemical profiles in females (**D**) and males (**E**)**.** Data are presented from 7 and 5 experiments in females and males, respectively. Notice, the difference in co-appearance of responses to 5 μM Yoda1 and 50 μM PregS in females and males. In females, up to 44% clusters were activated by PregS and Yoda1 (23%) or by PregS, Yoda1 and capsaicin (21%), whereas in males, the number of such ‘supermechanosensitive’ fibres was only 24% (PregS and Yoda1 (7%); PregS, Yoda1 and capsaicin (17%)). In males, 7% of clusters showed no response to any agonist, whereas this was the case for only 1% of clusters in females where, in addition, no fibres responded only to 5 μM Yoda1

To assess whether the activation of mechanosensitive Piezo1 and TRPM3 channels varies for single nerve fibres or small groups of fibres (clusters), we used a clustering approach [13]. Figure 5 A shows examples of multiple-spike clusters of experiments from female and male mice under control conditions and during 50 μM PregS application, whereby each dot represents one spike and different colours indicate different spike clusters. The data reveal that, especially in females, the TRPM3 channel agonist PregS increased the number of spikes in each of the identified clusters and additionally ‘wakes up’ previously silent clusters that consisted of spikes with a relatively large positive and negative amplitude. One advantage of the clustering approach is that it is able to shed light on whether different nociceptors are co-expressed within the same fibre, i.e. one cluster. Figure 5 Ba-d and Ca-d show example recordings illustrating the activity of selected clusters, indicating that different combinations of the two mechanosensitive Piezo1 and TRPM3 channels may occur within one cluster (fibre), as well as revealing whether a fibre responds to activation of classical nociceptors by capsaicin or not. Our functional data indicate that the latter are typically expressed in fibres with small- and prolonged-amplitude of spikes (Fig. 5 Ba,c and Ca,c), a signature of C-fibres [28] that are typically responsive to capsaicin [13]. Based on the joint responsivity to Yoda1 and PregS, Piezo1 channels are co-expressed with TRPM3 in capsaicin-sensitive (Fig. 5 Ba and Ca) as well as capsaicin-insensitive fibres (Fig. 5 Bb and Cb). Notably, TRPM3 channels could be activated in most (98% in females vs. 80% in males) fibres (including fibres that were insensitive to Yoda1 and/or capsaicin, see Fig. 5 Bb-d or Cb-d). Notably, the application of PregS caused a massive and repetitive escalation of spike frequency in clusters that had been silent throughout the whole application in females (Fig. 5 Bd), while in males (Fig. 5 Cd) in the same type of clusters PregS induced short peaks in firing rate. Thus, the different shapes of recorded spikes correlated to different types of activated fibres, based on the distinct responses to Yoda1, PregS, and capsaicin. Figure 5 D and E summarizes the neurochemical response profile of different meningeal fibres (represented by the distinct spike clusters) in females and males. Interestingly, in females, up to 44% clusters (63 out of 143) were activated by PregS and Yoda1 (23%) or by PregS and Yoda1 as well as capsaicin (21%), whereas in males the number of such ‘supermechanosensitive’ fibres was only 24% (17 out of 70; including PregS and Yoda1 (7%); PregS, Yoda1 and capsaicin (17%)). In contrast, the profile of ‘only-PregS-sensitive’ (46% in females vs. 44% in males) or all capsaicin-sensitive (30% in females vs. 33% in males) fibres did not show sex-dependence. In summary, spike cluster analysis revealed a prevailing functional activity of mechanosensitive TRPM3 channels in individual meningeal nerve fibres in females and, typical for this sex, massive and repetitive escalation of spike frequency in all clusters following pharmacological activation of these channels.

### Spectral analysis of the nociceptive spiking activity induced by TRPM3 activation

**Fig. 6:**
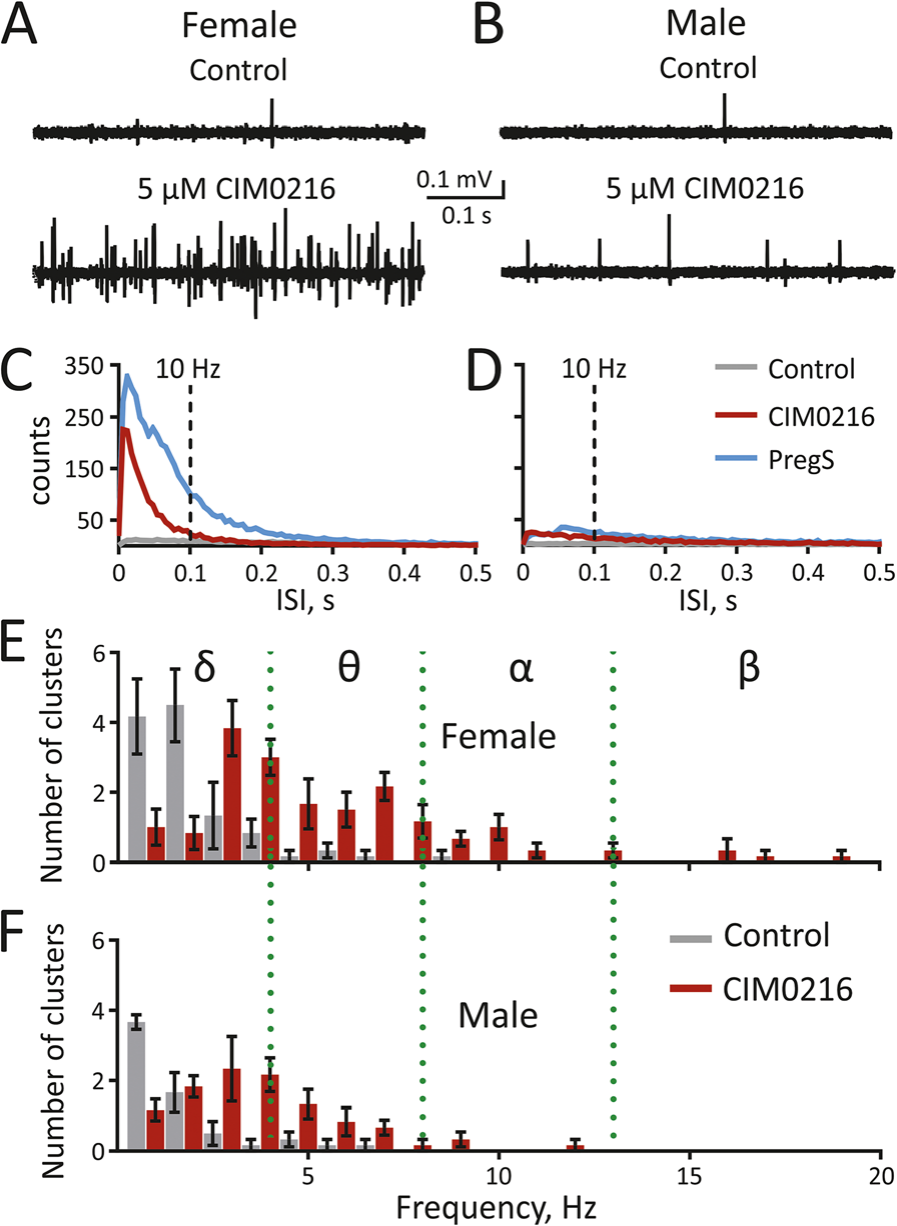
Spectral analysis of nociceptive firing in control conditions and the presence of CIM0216. (**A, B**) Example traces of MUA within one experiment in the control condition and during 5 μM CIM0216 in females (**A**) and males (**B**). Notice, there is only a single spike in the control condition in both males and females, while during the 2-min active phase of 5 μM CIM0216 application, the number of spikes increased differently for females and males, within the initial 0.5-s of drug application. **(C, D)** Spectrograms show the average interspike intervals (ISI) for all experiments in females (**C**) in the control conditions (grey line, *n* = 13), after application of 50 μM PregS (blue line, *n* = 7) and after 5 μM CIM0216 application (red line, *n* = 6) and in males (**D**) in the control condition (grey line, *n* = 11), after application of 50 μM PregS (blue line, *n* = 5) and after 5 μM CIM0216 application (red line, *n* = 6) on panel. The dotted vertical line at 0.1 ISI indicates the 10 Hz frequency. The number of ISIs below 0.1 s (i.e. reflecting spiking activity above 10 Hz frequency) is higher in females after 50 μM PregS and 5 μM CIM0216. (**E, F**) Distribution of fibres (clusters) according to the firing frequency in the control condition (top panel; grey) and after application of 5 μM CIM0216 (bottom panel; red) presented as mean ± SEM for females (**E**) (*n* = 6) and males (**F**) (*n* = 6) on panel (**F**). Notice that 5 μM CIM0216 induced nociceptive firing with θ-range of spiking activity for both sexes, whereas in females, θ-range, as well as higher spiking activity of α and β-ranges, were induced

Finally, we characterized the temporal patterns of spike activity in meningeal nerves induced by the TRPM3 channel agonists. Our spectral analysis showed that application of either CIM0216 or PregS remarkably increased nociceptive firing in the range of very brief interspike intervals (ISI), especially in females (examples of original traces in Fig. 6 A and B and averaged data in Fig. 6 C and D). Firing corresponding to the spiking activity in δ-range (1–4 Hz) was prevalent in the majority of fibres during control conditions with no difference between males (88 ± 5.9% of fibres, *n* = 6) and females (87.7 ± 5.6% of fibres, *n* = 6). In contrast, after application of 5 μM CIM0216, we observed additional spiking activity in the θ- and α-ranges (4–8 Hz and 8–13 Hz, respectively) in both sexes with a predominance in females, as well as β-range of spiking activity (13–20 Hz) only in females (Fig. 6 E, H). One can envisage that high-frequency firing induced by TRPM3 agonists is supposed to provide the temporal summation of nociceptive signalling at the level of second order nociceptive neurons in the brainstem [13, 25], thus facilitating nociceptive traffic to higher pain centers in the brain.

## Discussion

One of the main findings of our study is the demonstration of functional TRPM3 channels in peripheral meningeal terminals of the TG nerve. In addition, we could show that the spiking activity at terminals in response to activation of TRPM3 channels was higher in females than in males. Hence, we provided first experimental evidence of the presence and function of TRPM3 channels in nerves that are considered very relevant for migraine pathogenesis. Moreover, our spike cluster analysis revealed the diverse contribution of individual TG fibres to nociceptive firing induced by TRPM3 channel activation, with preferential induction of large-amplitude spikes in female mice. Spectral analysis also showed a difference between both sexes with respect to the prevailing spiking activity of nociceptive firing evoked by the CIM0216, namely θ- and α-ranges of spiking activity in females and δ- and θ-ranges in males. Taken together, our study suggests that activation of TRPM3 channels may be involved in triggering pain generation in meninges and seems to play a prominent role in mechanisms underlying sex differences in migraine pathology.

There is compelling evidence that activation of the meningeal trigeminovascular system through firing of peripheral nerve terminals is involved in initiating migraine headaches [13, 29]. Many molecules and signalling pathways are likely involved in detecting, transduction, and propagation of this nociceptive firing [30, 31]. One of the less-studied but interesting molecular sensors is the TRPM3 channel with its wide hormonal regulation profile and recently proposed role in nociceptive mechanisms [32]. In addition, TRPM3 channels are expressed in the peripheral nervous system, where they have been characterized as a noxious heat sensor in somatosensory neurons [15, 33, 34]. Although TRPM3 channels have been intensively studied in somatosensory neurons of DRGs and TG ganglia [15], they have not been considered as relevant to migraine pathophysiology and had not been identified in the meningeal afferents. Considering the rising spiking activity in meningeal afferents as a nociceptive effect [13, 35], we here provide first evidence of TRPM3 channels in relation to the site of origin of migraine pain.

Although we used PregS at a high micromolar concentration to obtain a robust activation of TG nerve terminals, there is already noticeable activation of TRPM3 channels in the somatosensory system at low nanomolar (physiological) concentrations [15]. This may suggest that PregS can sensitise nociceptive afferents for other potential triggers or trigger migraine-related nociceptive firing in meningeal afferents in conditions when the concentration of the endogenous steroid is increased, e.g. during parturition and under various pathological conditions [36]. Further support for the role of TRPM3 in meningeal nociception was obtained in our present study that revealed an exceptionally strong sex-dependent effect of synthetic agonist CIM0216 on the activation of TG fibres. It is worth mentioning, that PregS activated the TRPM3 channels during the entire 10 min of application, whereas 5 μM CIM0216 induced only a short-lasting activation followed by a block of all nociceptive activity. This silencing of firing after activation could be either due to desensitization of TRPM3 receptors as desensitization is a common agonist-specific mechanism shaping the duration of physiological responses or activation of calcium-dependent potassium conductance, which limits the depolarizing response in primary afferents. The latter obviously is stronger in the case of CIM0216, which triggers, more so than PregS, calcium influx via TRPM3 channels.

To disentangle nociceptive signalling caused by PregS in TG fibres, we used cluster analysis that can identify clusters (fibres) that simultaneously, but selectively, respond to more than one agonist or just one agonist. Our comparative analysis showed that the profile of ‘only-PregS-sensitive’ and ‘capsaicin-sensitive fibres’ did not reveal sex-dependence. Moreover, in both sexes, we found that activation of TRPM3 channels by PregS reveals clusters that had been silent before drug application. However, unlike in males, in females such clusters showed a massive and repetitive escalation of spike frequency in meningeal afferents. Furthermore, the ‘woken-up’ spike clusters consist of a larger amplitude and a faster time-course, which is a typical signature of Aδ fibres [28]. It is also worth mentioning that females and males differed with respect to the group of clusters that responded to both Yoda1 and PregS (23% vs. 7%), which reflects sex differences in the co-existence of the two types of mechanosensitive channels Piezo1 and TRPM3. In addition, cluster analysis indicated that, in females, the number of ‘super-mechanosensitive’ fibres (i.e. those expressing both TRPM3 and Piezo1 channels) was two times higher than in males. Taken together, the data reveal that, unlike Piezo1 and TRPV1 channels, the contribution of TRPM3 channels to nociception firing is different between sexes at the level of single fibres in meningeal afferents.

Mechanosensitive channels can be activated by a variety of mechanical or chemical stimuli [37], so it remains an enigma which of the stimuli play a role in the activation of meningeal afferents. There are several potential factors converting mechanical stimuli into electrical signals, such as osmotic swelling (proposed for TRPM3 channels [38]) and pulsatile blood flow in the meninges (proposed for Piezo1 channels [11]). Mechanically sensitive Piezo1 channels were recently identified in peripheral meningeal terminals of TG ganglia [11, 12]. However, data presented in the current study, for the first time, show the absence of a sex difference in the activation of Piezo1 channels in meningeal afferents that have been implicated in the pathogenesis of migraine headache.

The revealed absence of sex differences in the nociceptive responses to Piezo1 agonist Yoda1 or TRPV1 agonist capsaicin underscores the specific role of sex in the regulation of TRPM3 channels. Unlike Piezo1 channels, which can be activated by synthetic agonist Yoda1, TRPM3 channels in the case if they are activated by the endogenous agonist PregS, potentially could be suppressed by sex hormones progesterone and 17β-oestradiol [20]. In comparison with DHT, progesterone is more powerful and shows effects with EC50 in the range from 10 nM to 10 μM with retention of its impact in the absence of PregS. In their study, Majeed and co-authors stated that 17β-estradiol had much less or no inhibitory effect on TRPM3 channel activity. In clinical studies, effects of progesterone on TRPM3 channels were observed at the upper end of the physiologically relevant concentration range, with an EC50 of 1–2 nM, rising to 30–50 nM in the luteal phase of the menstrual cycle [39]. This increases the likelihood that TRPM3 channels are regulated by progesterone in vivo. Of note, it was also shown that 17β-estradiol increased neurogenic vasodilation in the dura mater, suggesting an increased release of CGRP from the perivascular nerves. This may be one mechanism by which 17β-estradiol exacerbates migraines in women [40]. Of relevance, it was shown that expression of the TRPM3 channel gene *Trpm3* increased during proestrus in mice, precisely when estrogen and progesterone levels change, i.e. estradiol rises within 12 h and peaks at about midday, and then, over the next 6 h drops to one fifth of the peak level [41]. The dramatic decrease in estradiol during proestrus co-incides with an increase in progesterone [42–44]. The intriguing role of female hormones in modulating the activity of TRPM3 channels deserves further exploration in in vivo models of migraine.

## Conclusion

This study, for the first time, demonstrates the presence of TRPM3 channels in the peripheral part of meningeal TG nerves that are considered relevant for migraine pathogenesis. Considering the broad hormonal regulation profile of TRPM3 channels and observing the significant difference in TRPM3 channel activity between males and females, we propose that TRPM3 channels play an important role in the mechanisms underlying sex differences in migraine. Therefore, taking into account the molecular and mechanosensitive properties of TRPM3 channels, we envisage TRPM3 channels as an attractive new target for drug interventions in migraine.

## Data Availability

The datasets used and/or analysed during the current study are available from the corresponding author on reasonable request.

## Abbreviations

aCSF: Artificial cerebrospinal fluid
CGRP: Calcitonin gene-related peptide
DHT: Dihydrotestosterone
ISI: Interspike intervals
MUA: Multi-unit activity
PregS: Pregnenolone sulfate
RT: Room temperature
SEM: Standard error of the mean
TRPM3: Transient receptor potential melastatin 3
TRPV1: Transient receptor potential vanilloid 1
TG: Trigeminal
WT: Wildtype
