# Correction to: Migraine-relevant sex-dependent activation of mouse meningeal afferents by TRPM3 agonists

**DOI:** 10.1186/s10194-022-01390-3

**Published:** 2022-01-28

**Authors:** G. Krivoshein, E. A. Tolner, A. M. J. M. van den Maagdenberg, R. A. Giniatullin

**Affiliations:** 1 A.I.Virtanen Institute for Molecular Sciences, University of Eastern Finland, Kuopio, Finland; 2 Department of Human Genetics, Leiden University Medical Center, Leiden, The Netherlands; 3 Department of Neurology, Leiden University Medical Center, Leiden, The Netherlands; 4 Laboratory of Neurobiology, Kazan Federal University, Kazan, Russia

**Correction to: J Headache Pain 23, 4 (2022)**

**https://doi.org/10.1186/s10194-021-01383-8**

Following the publication of the original article [1], we were notified that the first and last name of AMJM van den Maagdenberg had been swapped.

Originally published name: van den Maagdenberg AMJM

Corrected name: AMJM van den Maagdenberg

The original article has been corrected.
